# Risk of cardiovascular event and mortality in relation to refill and guideline adherence to lipid-lowering medications among patients with type 2 diabetes mellitus in Sweden

**DOI:** 10.1136/bmjdrc-2018-000639

**Published:** 2019-04-08

**Authors:** Sofia Axia Karlsson, Björn Eliasson, Stefan Franzén, Mervete Miftaraj, Ann-Marie Svensson, Karolina Andersson Sundell

**Affiliations:** 1 Department of Public Health and Community Medicine, Institute of Medicine, Sahlgrenska academy, University of Gothenburg, Goteborg, Sweden; 2 Department of Molecular and Clinical Medicine, Institute of Medicine, Sahlgrenska Academy, University of Gothenburg, Gothenburg, Sweden; 3 National Diabetes Register, Centre of Registers, Gothenburg, Sweden; 4 Global Medical Affairs, Medical Evidence and Observational Research, AstraZeneca AB, Mölndal, Sweden

**Keywords:** refill adherence, guideline adherence, type 2 diabetes mellitus, lipid-lowering medications, cardiovascular disease prevention, cardiovascular event, cardiovascular disease

## Abstract

**Objective:**

To analyze the risk of cardiovascular (CV) events and mortality in relation to adherence to lipid-lowering medications by healthcare centers and patients with type 2 diabetes mellitus (T2DM).

**Research design and methods:**

We included 121 914 patients (12% secondary prevention) with T2DM reported by 1363 healthcare centers. Patients initiated lipid-lowering medications between July 2006 and December 2012 and were followed from cessation of the first filled supply until multidose dispensed medications, migration, CV events, death or December 2016. The study period was divided into 4-month intervals through 2014, followed by annual intervals through 2016. Adherence measures were assessed for each interval. Patients’ (refill) adherence was measured using the medication possession ratio (MPR). Healthcare centers’ (guideline) adherence represented the prescription prevalence of lipid-lowering medications according to guidelines. The risk of CV events and mortality was analyzed for each interval using Cox proportional hazard regression and Kaplan-Meier.

**Results:**

Compared with high-adherent patients (MPR >80%), low-adherent primary prevention patients (MPR ≤80%) showed higher risk of all outcomes: 44%–51 % for CV events, doubled for all-cause mortality and 79%–90% for CV mortality. Corresponding risks for low-adherent secondary prevention patients were 17%–19% for CV events, 88%–97% for all-cause and 66%–79% for CV mortality. Primary prevention patients treated by low-adherent healthcare centers (guideline adherence <48%) had a higher risk of CV events and CV mortality. Otherwise, no difference in the risk of CV events or mortality was observed by guideline adherence level.

**Conclusions:**

Our results demonstrate the importance of high refill adherence and thus the value of individualized care among patients with T2DM.

**Significance of this study**

**What is already known about this subject?**

- High refill adherence to lipid-lowering medications associates with lower risk of cardiovascular event and mortality among patients with type 2 diabetes mellitus; the impact of the treating healthcare centers’ adherence to lipid-lowering guidelines is uncertain.

**What are the new findings?**

- We found patients’ refill adherence to lipid-lowering medications to have greater impact than adherence to guidelines by the treating healthcare center in terms of prevention of cardiovascular event and mortality in both primary and secondary prevention patients.

**How might these results change the focus of research or clinical practice?**

- Our results emphasize the value of individualized care and the importance of maximizing refill adherence to lipid-lowering medications among patients with type 2 diabetes mellitus.

## Introduction

Type 2 diabetes mellitus (T2DM) is a multifactorial disease that requires intensive glycemic control and treatment for comorbidities and complications to reduce the increased risk of cardiovascular disease (CVD) and mortality.^1–3^ Despite declining mortality rates among Swedish patients with T2DM, the risk is still higher than among patients without diabetes mellitus, and CVD remains the major cause of death.^4^ Improved control of low-density lipoprotein (LDL) cholesterol with lipid-lowering medications has been shown to reduce the risk of CVD and mortality among patients with T2DM.^5–8^ Thus, national^9^ and international^1 10^ treatment guidelines recommended lipid-lowering medications to patients with T2DM to lower LDL cholesterol below 2.5 mmol/L. For patients with established CVD, LDL cholesterol below 1.8 mmol/L is desirable.

Adherence is defined as the extent to which individuals follow agreed recommendations.^11^ Previous studies have shown that patients with T2DM with adherence of at least 80% to lipid-lowering medications face a lower risk of CVD and mortality than those with adherence below 80%.^5 6 8 12 13^ Among multiple levels of adherence to lipid-lowering medications, a gradual increase in CVD risk was observed with declining adherence by patients with T2DM.^6^

Previous studies have reported adherence by healthcare providers to recommended treatment guidelines at 24%–80% among patients with diabetes mellitus.^14–19^ Patients with T2DM or established CVD were more likely to receive lipid-lowering medications. However, little is known about the impact of healthcare provider adherence to lipid-lowering medications on the risk of cardiovascular (CV) events or mortality among patients with T2DM.

Our primary objective was to analyze the risk of CV events and mortality in relation to T2DM patients' adherence to lipid-lowering medications and providers' adherence to lipid-lowering guidelines. Our secondary objective was to identify differences in the risk of CV events and mortality among various patient characteristics.

## Methods

### Data sources

The unique Swedish personal identity number was used to link individual-based data from six national registers. Filled prescriptions were collected from the Swedish Prescribed Drug Register (SPDR), which contains information about all prescriptions filled at Swedish pharmacies since July 2005.^20^ Swedish prescriptions include information about the medication, patient, prescriber and the prescribed daily dosage. Clinical and health-related data about risk factors and complications of diabetes, as well as healthcare center characteristics, were collected from the Swedish National Diabetes Register (NDR), which contains patient information reported by physicians and nurses at hospitals and primary care clinics nationwide.^21^ Healthcare providers were identified at the center level, not by individual practitioners. Data concerning CV events were collected from the Swedish National Patient Register, which contains information about inpatient and outpatient care in Sweden.^22^ The date and cause of death were collected from the Cause of Death Register.^23^ Information about primary tumors was collected from the Swedish Cancer Registry.^24^ Individual data about socioeconomic status were collected from the Longitudinal Integration Database for Health Insurance and Labour Market Studies (LISA).^25^

### Study population and period

**Figure 1:**
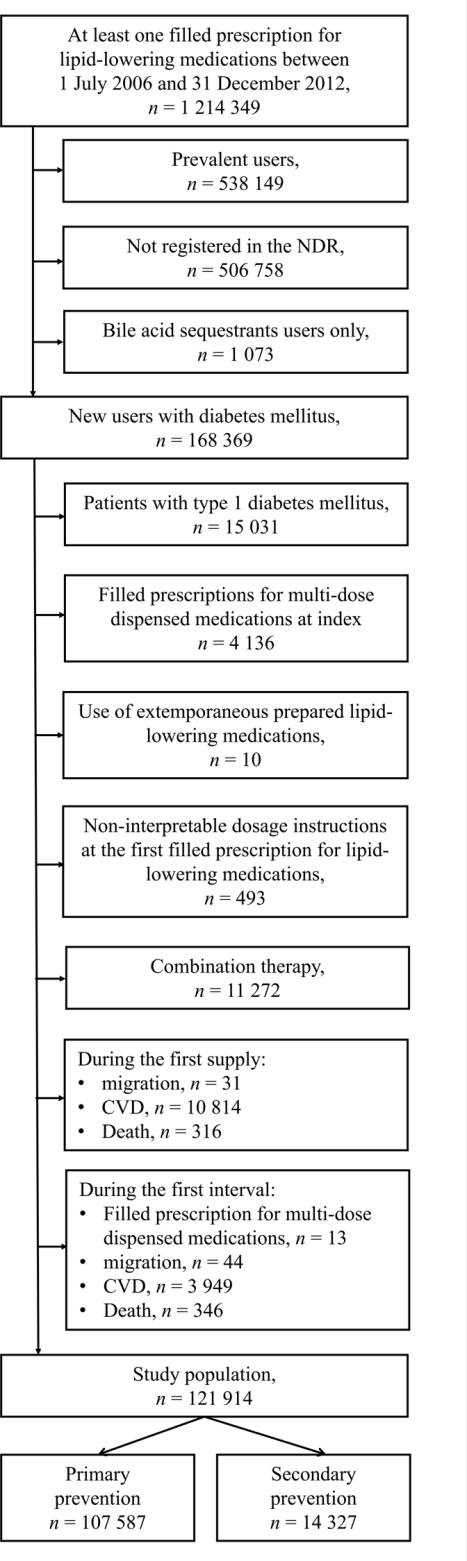
Inclusion and exclusion of the study population. CVD, cardiovascular disease; NDR, National Diabetes Register.

Patients age 18 years or older with a clinical T2DM diagnosis^26^ who had filled at least one prescription for lipid-lowering medications between July 1, 2006 and December 31, 2012 were eligible for inclusion (figure 1). Prescriptions for bile acid sequestrants were excluded since the indication is typically not hyperlipidemia.^27^ The date of the first filled prescription was defined as the index date. To establish a new-user design, patients who had filled prescriptions for lipid-lowering medications within 1 year prior to the index date were regarded as prevalent users and excluded. Patients who filled prescriptions for a lipid-lowering combination therapy or lipid-lowering extemporaneous preparation that lacked information about package size were also excluded. Combination therapy has been described elsewhere.^6 28^ Patients who experienced CVD prior to or on the index date were classified as prescribed lipid-lowering secondary prevention; all other patients were defined as primary prevention.

Patients were followed from cessation of the first filled supply (baseline date) until the first filled prescription for multi-dose dispensed medications, migration, CV event, death or December 31, 2016. The study period was broken down into subsequent intervals of 122 days until December 31, 2014, followed by annual intervals until December 31, 2016.

The healthcare center at baseline was assigned by selecting the entry in the NDR closest to the baseline date and remained the same throughout the study period unless the NDR indicated otherwise. A comparison between the assigned healthcare center from the NDR with the healthcare provider of the first filled lipid-lowering prescription in the SPDR showed agreement of 98% for county council and 87% for type of care.

### Refill adherence

Patients’ (refill) adherence to lipid-lowering medications was assessed with data from the SPDR. We assumed that patients initiated medication use on the index date. We measured refill adherence with the medication possession ratio (MPR), representing the proportion of days with medications available. The supply of each prescription was calculated by dividing the number of tablets filled by the daily dosage stated as a free-text variable by the prescriber. Interpretation of the free-text variable to obtain the daily dosage has been described elsewhere.^6 28^ Overlapping supplies for the same substance and strength were added to the latter supply. Overlapping supplies for different substances or strengths were deleted. MPR was assessed for each subsequent interval and categorized as high or low based on the common cut-off of 80%.^29 30^

### Guideline adherence

Based on data from the NDR, we assessed healthcare center adherence to national lipid-lowering prescription guidelines. Guideline adherence was defined as the prescription prevalence of lipid-lowering medications among patients with T2DM and LDL cholesterol above the recommended target levels existing at the time for the study (≥2.5 mmol/L for primary prevention^31^ and ≥1.8 mmol/L for secondary prevention^1 10^). Between 2007 and 2014, guideline adherence was assessed for each healthcare center and year, for primary and secondary prevention patients separately.

Guideline adherence was linked to patient intervals based on the year in which the interval started. For intervals starting in 2006, guideline adherence for 2007 was used. For healthcare centers that lacked information about adherence, we imputed the preceding year’s figure or the mean annual adherence for the county council and type of care. Guideline adherence was categorized as high or low based on a cut-off that represented the median for primary (48%) and secondary prevention (78%).

### Outcomes

The outcomes of interest were CV events and mortality. A CV event was defined as a diagnosis of unstable angina pectoris, myocardial infarction (including percutaneous coronary intervention, coronary artery bypass grafting), stroke or ischemic heart disease. All-cause mortality was defined as death from any cause. CV mortality was defined as death from a cause of CVD or a CV event entered in the National Patient Register within 28 days prior to death. Starting from the second interval, the risk of CV events and mortality was analyzed for each interval until migration, CV event, death or December 31, 2016.

### Covariates

Covariates regarded as potential confounders included sex, age, socioeconomic status (country of birth, marital status, education level, employment status, profession, income), concurrent medications (filled prescriptions for diabetes medications, anticoagulants and antihypertensives), and clinical and health-related characteristics (diabetes duration, hemoglobin A1c [HbA1c], estimated glomerular filtration rate [eGFR], blood pressure, cholesterol values, microalbuminuria, macroalbuminuria, kidney disease, cancer, body mass index [BMI], physical activity and smoking). These covariates have previously been shown to be important factors when analyzing adherence, as well as the risk of CV events and mortality.^6 28^

Sex, age and socioeconomic characteristics were collected from the LISA database. Sex and country of birth were regarded as constant, and age was based on the year of birth. Information about marital status, education level, employment status, profession and income was collected prior to or during the baseline year. Income was regarded as a continuous variable. The remaining socioeconomic variables included the following categories: country of birth: Sweden, other Nordic country, other European Union (EU)-15 country or the Soviet Union, rest of Europe, the Americas, Asia or Oceania, or unknown; marital status: unmarried, married or registered partner, divorced, or widow/widower; education level: compulsory school or lower, upper secondary school, or postsecondary; employment status: unemployed, employed or retired; profession: upper white-collar, lower white-collar, blue-collar, or other.

At baseline, cancer and kidney disease were defined as diagnosis within 5 years prior to the baseline date and were collected from the Swedish Cancer Registry and the National Patient Register, respectively. Cancer diagnosis included primary tumors, while kidney disease included acute or chronic kidney failure, as well as glomerular or renal complication due to T2DM.

Filled prescriptions for diabetes medications (anatomical therapeutic chemical (ATC): A10), anticoagulants (ATC: B01), and antihypertensives (ATC: C02, C03, C04, C05, C07, C08, and C09) were collected from the SPDR. Filled prescriptions within 12 months prior to the baseline date were considered concurrent use.

The remaining clinical and health-related characteristics were collected from the NDR. At baseline, data were collected between 24 months prior to and 14 days after the baseline date by selecting the value closest to baseline. Diabetes duration was based on the year of birth and diabetes diagnosis. Microalbuminuria and macroalbuminuria were dichotomously categorized. BMI and eGFR were categorized according to recommended references values.^32 33^ HbA1c, blood pressure and cholesterol levels were categorized as high or low according to recommended target values at the time of the study.^31^ Physical activity was defined as a 30 min walk or equivalent, categorized as less than once a week, 1–2 times a week, 3–5 times a week or daily. Smoking was dichotomized and defined as at least one cigarette/pipe daily or having quit within the past 3 months.

A total of 23% of patient characteristics at baseline were missing: 4% of socioeconomic status and 43% of clinical and health-related characteristics. No information was missing about age, sex or concurrent medications. Missing information at baseline was replaced with multiple imputations. Potential confounders (except for cancer) were assessed for each interval during the study period by assuming the imputed baseline value until the information had been updated in the registers.

### Sensitivity analyses

To evaluate the cut-offs used to categorize refill and guideline adherence as high or low, new ones were set. For refill adherence, cut-offs of 60%, 70%, and 90% were applied to evaluate the 80% level that had been used to define patients as low-adherent or high-adherent. For guideline adherence, new cut-offs were set at 30% and 60% (from 48%) for primary prevention and 50% and 90% (from 78%) for secondary prevention. The results of the statistical analyses were compared between the cut-offs.

To estimate the impact of multiple imputations, the risk of CVD and mortality was assessed and compared between complete cases and imputed data.

### Statistical analyses

The association between refill (MPR) and guideline adherence was analyzed by means of general linear regression. Multivariate imputations by chained equations (MICE) were used to replace missing information among baseline variables; 10 imputed data sets with 10 iterations each were generated. The risk of CV events and mortality was analyzed for each interval based on the 10 imputed data sets with Cox proportional hazard regression and Kaplan-Meier, adjusted for potential confounders. Covariates and guideline adherence for one interval were regarded as potential confounders for the subsequent interval of MPR measures. Similarly, MPR for one interval was regarded as the exposure for the subsequent interval of outcome measures (online supplementary figure S1). HRs generated for each imputed data set were pooled and one final set was established. The same procedure was performed to assess adjusted and pooled survival estimates and obtain Kaplan-Meier survival curves for CV events and mortality. All hypothesis tests were evaluated at a 5% significance level.

To present baseline characteristics for the study population based on imputed data, the mean values of continuous variables and the most frequent category of categorical variables were obtained from the imputed data sets. These generated baseline values were used to descriptively present the study population but were not used in the statistical analyses.

Multiple imputations were performed in R V.3.3.2^34^ using the MICE package.^35^ All other data management and statistical analyses were performed with SAS V.9.4 software.

## Results

### Study population and period

A total of 121 914 patients with T2DM were included (figure 1). Of them, a total of 11.8% started on lipid-lowering secondary prevention. The mean age was 63 years, 57% were men and the mean diabetes duration was 5 years (table 1). Approximately 80% were born in Sweden and more than 50% were married. Among primary prevention patients, the mean age was 62 years, 56% were men and the mean diabetes duration was 5 years. Among secondary prevention patients, the mean age was 70 years, 61% were men and the mean diabetes duration was 6.5 years.

**Table 1:** Baseline characteristics (imputed data) of all 121 914 new users of lipid-lowering medications with type 2 diabetes mellitus and by prevention type

		Total population, n=121 914	Primary prevention, n=107 587	Secondary prevention, n=14 327
		n (%)	n (%)	n (%)
Demographic and socioeconomic status				
Sex	Male	68 828 (56.5)	60 043 (55.8)	8785 (61.3)
Age (years)	18–40	3230 (2.7)	3187 (3.0)	43 (0.3)
	41–60	42 954 (35.2)	40 466 (37.7)	2388 (16.7)
	61–80	68 893 (56.5)	59 406 (55.2)	9487 (66.2)
	>80	6837 (5.6)	4428 (4.1)	2409 (16.8)
	Mean (SD)	63.3 (11.2)	62.4 (11.1)	70.3 (10.2)
	Median (IQR)	64.0 (15.0)	63.0 (15.0)	71.0 (14.0)
Country of birth	Sweden	96 174 (78.9)	84 645 (78.7)	11 529 (80.5)
Other Nordic country	6761 (5.6)	5786 (5.4)	975 (6.8)
	Other European Union-15* country	2097 (1.7)	1827 (1.7)	270 (1.9)
	Other European country/Soviet Union	5874 (4.8)	5143 (4.8)	731 (5.1)
	Africa	1850 (1.5)	1748 (1.6)	102 (0.7)
	The Americas	1285 (1.1)	1171 (1.1)	114 (0.8)
	Asia/Oceania	7715 (6.3)	7120 (6.6)	595 (4.2)
	Unknown	158 (0.1)	147 (0.1)	11 (0.1)
Marital status	Unmarried	20 024 (16.4)	18 398 (17.1)	1626 (11.4)
	Married/Registered partner	66 760 (54.8)	59 306 (55.1)	7454 (52.0)
	Divorced	22 258 (18.3)	19 502 (18.1)	2756 (19.2)
	Widow/Widower	12 872 (10.6)	10 381 (9.7)	2491 (17.4)
Education level	Compulsory school or lower	46 718 (38.3)	39 773 (37.0)	6945 (48.5)
	Upper secondary school	53 717 (44.1)	48 222 (44.8)	5495 (38.4)
	Postsecondary	21 479 (17.6)	19 592 (18.2)	1887 (13.2)
Employment status	Unemployed	17 661 (14.5)	15 990 (14.9)	1671 (11.7)
Employed	59 290 (48.6)	55 232 (51.3)	4058 (28.3)
	Retired†	44 963 (36.9)	36 365 (33.8)	8598 (60.0)
Profession	Upper white-collar	32 535 (26.7)	29 145 (27.1)	3390 (23.7)
	Lower white-collar	10 232 (8.4)	9207 (8.6)	1025 (7.2)
	Blue-collar	75 708 (62.1)	66 308 (61.6)	9400 (65.6)
	Others	3439 (2.8)	2927 (2.7)	512 (3.6)
Income (thousand Swedish krona)	Per household member, mean (SD)	214.3 (429.2)	216.9 (422.0)	195.3 (478.8)
Per household member, median (IQR)	180.0 (117.0)	185.0 (120.0)	156.5 (84.9)
Concurrent medications				
Diabetes medications	Any	80 889 (66.4)	72 126 (67.0)	8763 (61.2)
Anticoagulants	Any	48 138 (39.5)	35 387 (32.9)	12 751 (89.0)
Antihypertensives	Any	90 160 (74.0)	76 996 (71.6)	12 164 (91.9)
Clinical and health-related characteristics				
Diabetes duration (years)	Mean (SD)	5.0 (6.4)	4.8 (6.1)	6.5 (7.7)
Median (IQR)	3.0 (8.0)	2.2 (7.0)	4.0 (10.0)
HbA1c, mmol/mol (%)	<42 (<5)	9706 (8.0)	8754 (8.1)	952 (6.6)
42–52 (5–6)	61 162 (50.2)	53 525 (49.8)	7637 (53.3)
	>52 (>6)	51 046 (41.9)	45 308 (42.1)	5738 (40.1)
	Mean (SD)	53.0 (12.0)	53.1 (12.1)	52.4 (10.9)
	Median (IQR)	50.0 (12.6)	50.0 (12.8)	50.0 (12.0)
eGFR	<60	9606 (7.9)	7521 (7.0)	2085 (14.6)
(mL/min/1.73 m†)	≥60	112 308 (92.1)	100 066 (93.0)	12 242 (85.5)
	Mean (SD)	85.3 (20.3)	86.3 (20.2)	77.5 (18.9)
	Median (IQR)	84.3 (22.6)	85.3 (22.4)	77.4 (21.1)
BMI	<18.5	173 (0.1)	155 (0.1)	18 (0.1)
(kg/m†)	18.5–24.9	10 015 (8.2)	8795 (8.2)	1220 (8.5)
	25.0–29.9	52 074 (42.7)	45 169 (42.0)	6905 (48.2)
	≥30.0	59 652 (48.9)	53 468 (49.7)	6184 (43.2)
	Mean (SD)	30.3 (4.5)	30.4 (4.5)	29.8 (4.2)
	Median (IQR)	29.9 (4.8)	30.0 (4.9)	29.5 (4.2)
Systolic pressure (mm Hg)	<130	27 429 (22.5)	25 080 (46.3)	2349 (16.4)
≥130	94 485 (77.5)	57 826 (53.8)	11 978 (83.6)
	Mean (SD)	137.6 (13.7)	137.4 (13.7)	139.5 (13.7)
	Median (IQR)	137.3 (14.2)	136.8 (14.0)	139.9 (13.4)
Diastolic pressure (mm Hg)	<80	58 138 (47.7)	49 761 (46.3)	8377 (58.5)
≥80	63 776 (52.3)	57 826 (53.8)	5950 (41.5)
	Mean (SD)	79.1 (8.0)	79.3 (8.0)	77.4 (7.8)
	Median (IQR)	80.0 (8.0)	80.0 (8.5)	78.2 (7.2)
LDL cholesterol (mmol/L)	<2.5	11 556 (9.5)	9937 (9.2)	1619 (11.3)
≥2.5	110 358 (90.5)	97 650 (90.8)	12 708 (88.7)
	Mean (SD)	3.4 (0.7)	3.4 (0.7)	3.2 (0.7)
	Median (IQR)	3.4 (0.7)	3.4 (0.7)	3.3 (0.7)
HDL cholesterol (mmol/L)	<1.0 (men)/<1.3 (women)	30 489 (25.0)	27 425 (25.5)	3064 (21.4)
≥1.0 (men)/≥1.3 (women)	91 425 (75.0)	80 162 (74.5)	11 263 (78.6)
	Mean (SD), men/women	1.2 (0.3)/1.4 (0.3)	1.2 (0.3)/1.4 (0.2)	1.2 (0.2)/1.4 (0.3)
	Median (IQR), men/women	1.2 (0.3)/1.3 (0.3)	1.1 (0.3)/1.4 (0.3)	1.2 (0.3)/1.4 (0.3)
Triglycerides (mmol/L)	<2.0	73 909 (60.6)	64 400 (59.9)	9509 (66.4)
≥2.0	48 005 (39.4)	43 187 (40.1)	4818 (33.6)
	Mean (SD)	2.0 (0.7)	2.0 (1.1)	1.9 (0.9)
	Median (IQR)	1.8 (0.9)	1.8 (0.9)	1.7 (0.7)
Microalbuminuria	Yes	10 693 (8.8)	9138 (8.5)	1555 (10.9)
Macroalbuminuria	Yes	3890 (3.2)	3222 (3.0)	668 (4.7)
Kidney disease	Yes	1859 (1.5)	1412 (1.3)	447 (3.1)
Cancer diagnosis	Yes	4521 (3.7)	3817 (3.6)	704 (4.9)
Physical activity‡	Less than once per week	21 201 (17.4)	18 071 (16.8)	3130 (21.9)
	1–2 times/week	23 635 (19.4)	21 017 (19.5)	2618 (18.3)
	3–5 times/week	29 670 (24.3)	26 803 (24.9)	2867 (20.0)
	Daily	47 408 (38.9)	41 696 (38.8)	5712 (39.9)
Smoking§	Yes	13 718 (11.3)	12 581 (11.7)	1137 (7.9)

*Includes Belgium, Denmark, Germany, Greece, Spain, France, Ireland, Italy, Luxembourg, The Netherlands, Austria, Portugal, Finland, Sweden and Great Britain.

†If age 65 years or older and unemployed.

‡30 min walk or equivalent.

§At least one cigarette or pipe daily or quit smoking within 3 months.

BMI, body mass index;HDL, high-density lipoprotein;HbA1c, hemoglobin A1c;LDL, low-density lipoprotein;eGFR, estimated glomerular filtration rate.

A total of 67% of primary prevention patients filled prescriptions for diabetes medications, 33% for anticoagulants and 72% for antihypertensives. A total of 61% of secondary prevention patients filled prescriptions for diabetes medications, 89% for anticoagulants and 92% for antihypertensives. Cancer and kidney disease prior to the index date had been diagnosed in 4% and 1% of primary prevention patients, respectively, and 5% and 3% of secondary prevention patients, respectively. A total of 17% of primary prevention patients were physically active less than once a week and 12% were smokers. The corresponding figures for secondary prevention patients were 22% and 8%. For both prevention groups, the mean HbA1c was above 52 mmol/mol, the mean BMI was approximately 30 and the mean LDL cholesterol was above 3 mmol/L.

The mean supply for the first filled prescription was 87 (±28) days for primary prevention patients and 90 (±25) days for secondary prevention patients. Among primary prevention patients, the mean study period was 2389 (±855) days for CVD and 2545 (±761) for mortality. Among secondary prevention patients, the mean study period was 1690 (±1071) days for CVD and 2416 (±885) days for mortality. A total of 79% (n=84 562) of primary prevention patients were not censored or experienced any outcome during the study period and were thus followed until December 31, 2016. The corresponding figure for secondary prevention patients was 38% (n=5413).

For CVD measures a total of 1% (n=1095) of primary prevention patients and 8% (n=1115) of secondary prevention patients were followed through one interval of outcome. For mortality measures, less than 1% of all patients were followed through one interval only.

### Refill and guideline adherence

Among patients with primary prevention, the overall mean refill adherence (MPR) was 63.6%, ranging from 60.7% to 70.0% throughout the study period. The mean refill adherence (MPR) for secondary prevention patients ranged from 55.6% to 73.1% throughout the study period, averaging 61.2%. A total of 6% of primary prevention patients and 10% of secondary prevention patients filled only their initial prescription.

A total of 1363 healthcare centers, 93% of which were primary care, were included. The overall mean annual guideline adherence was 49%, ranging from 48% to 49% throughout the study period for primary prevention patients, and 76%, ranging from 73% to 77% throughout the study period for secondary prevention patients.

With each percentage of guideline adherence, refill adherence increased by 0.01% for primary prevention patients and 0.002% for secondary prevention patients (p<0.0001).

### CV events and mortality

During the study period, 21 396 patients experienced CV events and 17 282 died. Almost 33% of all deaths were caused by CVD. A total of 13% of primary prevention patients experienced CV events and 12% died during the study period. A total of 51% of secondary prevention patients experienced CV events and 32% died during the study period. Death due to CVD accounted for 28% and 47% of all deaths among primary and secondary prevention patients, respectively.

Adjusted for potential confounders, primary prevention patients with low refill adherence had a 44%–51% higher risk of CV events, and 79%–92% higher risk of CV mortality and doubled risk of all-cause mortality (table 2). Primary prevention patients with low refill adherence who were being treated by healthcare centers with low guideline adherence faced an approximately 9% higher risk of CV events. Primary prevention patients who were being treated by healthcare centers with low guideline adherence had a 10%–18% higher risk of CV mortality.

**Table 2:** Risk of CV events and mortality by refill and guideline adherence level

			CV event		All-cause mortality		CV mortality	
	Refill adherence	Guideline adherence	HR (95% CI)		HR (95% CI)		HR (95% CI)	
			Crude	Adjusted*	Crude	Adjusted*	Crude	Adjusted*
Primary prevention, n=107 587	Low vs high	Low	1.56 (1.49 to 1.63)	1.51 (1.44 to 1.59)	2.25 (2.14 to 2.36)	2.05 (1.95 to 2.16)	2.18 (1.99 to 2.39)	1.92 (1.74 to 2.11)
	Low vs high	High	1.49 (1.41 to 1.56)	1.44 (1.37 to 1.51)	2.22 (2.11 to 2.34)	2.01 (1.90 to 2.12)	2.08 (1.88 to 2.30)	1.79 (1.61 to 1.99)
	Low	Low vs high	1.08 (1.03 to 1.13)	1.09 (1.04 to 1.14)	1.00 (0.96 to 1.05)	1.05 (1.00 to 1.09)	1.13 (1.03 to 1.22)	1.18 (1.08 to 1.28)
	High	Low vs high	1.03 (0.98 to 1.08)	1.03 (0.98 to 1.08)	0.99 (0.94 to 1.05)	1.02 (0.97 to 1.09)	1.07 (0.96 to 1.19)	1.10 (0.99 to 1.23)
Secondary prevention, n=14 327	Low vs high	Low	1.21 (1.14 to 1.29)	1.19 (1.11 to 1.27)	2.26 (2.07 to 2.46)	1.88 (1.73 to 2.06)	2.03 (1.80 to 2.30)	1.66 (1.46 to 1.89)
	Low vs high	High	1.21 (1.13 to 1.30)	1.17 (1.10 to 1.26)	2.37 (2.17 to 2.59)	1.97 (1.80 to 2.15)	2.20 (1.94 to 2.50)	1.79 (1.57 to 2.03)
	Low	Low vs high	1.00 (0.94 to 1.07)	1.01 (0.94 to 1.08)	0.98 (0.91 to 1.05)	0.99 (0.92 to 1.06)	0.95 (0.85 to 1.06)	0.95 (0.86 to 1.06)
	High	Low vs high	1.00 (0.94 to 1.07)	1.00 (0.94 to 1.06)	1.03 (0.93 to 1.14)	1.03 (0.93 to 1.14)	1.03 (0.89 to 1.18)	1.03 (0.89 to 1.18)

Crude and adjusted HR (with 95% CI) for any CV events, all-cause mortality and CV mortality among patients with type 2 diabetes mellitus by refill and guideline adherence level, as well as prevention type.

*Adjusted for age, sex, socioeconomic status, concurrent medications, as well as clinical and health-related characteristics.

CV, cardiovascular.

Secondary prevention patients with low refill adherence had a 17%–19% higher risk of CV events, 88%–97% higher risk of all-cause mortality and 66%–79% higher risk of CV mortality than those with high refill adherence (table 2). There was no statistically significant difference in the risk of CV events and mortality between guideline adherence levels among secondary prevention patients.

In general, the risk of CV events and mortality was higher with increasing age, diabetes duration and HbA1c, as well as among patients with low kidney function (kidney disease, microalbuminuria or macroalbuminuria), physical activity less than twice a week, smokers and men (table 3). A lower risk was observed among patients who had not filled prescriptions for anticoagulants or antihypertensives, as well as among patients born outside of Sweden.

**Table 3:** Risk of CV events and mortality for patient characteristics

	Primary prevention, n=107 587			Secondary prevention, n=14 327		
	CV events	All-cause mortality	CV mortality	CV events	All-cause mortality	CV mortality
	HR (95% CI)	HR (95% CI)	HR (95% CI)	HR (95% CI)	HR (95% CI)	HR (95% CI)
Sex, ref=female						
Male	1.54 (1.48 to 1.60)	1.43 (1.37 to 1.49)	1.62 (1.50 to 1.75)	1.35 (1.28 to 1.42)	1.48 (1.38 to 1.59)	1.62 (1.46 to 1.80)
Age (years), ref=61–80						
18–60	0.61 (0.58 to 0.65)	0.45 (0.42 to 0.49)	0.45 (0.38 to 0.52)	0.95 (0.87 to 1.05)	0.50 (0.41 to 0.61)	0.48 (0.36 to 0.65)
>80	1.46 (1.39 to 1.54)	2.19 (2.09 to 2.29)	2.32 (2.13 to 2.52)	1.35 (1.27 to 1.43)	2.13 (1.99 to 2.29)	2.35 (2.12 to 2.61)
Country of birth, ref=Sweden						
Other Nordic country	1.18 (1.11 to 1.27)	1.02 (0.95 to 1.10)	1.10 (0.96 to 1.25)	1.14 (1.04 to 1.25)	0.99 (0.88 to 1.12)	1.06 (0.89 to 1.26)
Other European Union-15* country	0.89 (0.78 to 1.01)	0.82 (0.72 to 0.94)	0.77 (0.59 to 1.01)	1.18 (1.00 to 1.39)	1.07 (0.87 to 1.32)	1.22 (0.91 to 1.63)
Other European country/Soviet Union	1.11 (1.03 to 1.20)	0.89 (0.81 to 0.97)	0.86 (0.72 to 1.03)	1.08 (0.97 to 1.21)	0.86 (0.74 to 1.00)	0.84 (0.67 to 1.05)
Africa	0.68 (0.57 to 0.81)	0.47 (0.37 to 0.61)	0.41 (0.24 to 0.69)	0.97 (0.72 to 1.30)	0.92 (0.59 to 1.43)	1.02 (0.54 to 1.90)
The Americas	0.84 (0.69 to 1.01)	0.64 (0.51 to 0.81)	0.64 (0.41 to 1.00)	0.70 (0.52 to 0.96)	0.88 (0.60 to 1.30)	0.73 (0.39 to 1.37)
Asia/Oceania	1.15 (1.07 to 1.25)	0.48 (0.43 to 0.54)	0.63 (0.51 to 0.77)	1.15 (1.02 to 1.30)	0.71 (0.58 to 0.87)	0.74 (0.55 to 1.00)
Unknown	1.38 (0.91 to 2.11)	0.82 (0.44 to 1.54)	0.89 (0.29 to 2.79)	1.47 (0.70 to 3.10)	NA	NA
Marital status, ref=married						
Unmarried	0.87 (0.83 to 0.92)	1.18 (1.12 to 1.25)	1.41 (1.27 to 1.57)	0.89 (0.82 to 0.97)	1.26 (1.13 to 1.40)	1.46 (1.26 to 1.69)
Divorced	1.04 (0.99 to 1.09)	1.11 (1.05 to 1.17)	1.17 (1.06 to 1.30)	1.01 (0.95 to 1.08)	1.05 (0.96 to 1.14)	1.06 (0.93 to 1.21)
Widow(er)	1.14 (1.09 to 1.20)	1.20 (1.14 to 1.26)	1.38 (1.25 to 1.51)	1.06 (0.99 to 1.13)	1.20 (1.11 to 1.29)	1.19 (1.06 to 1.33)
Education level, ref=mandatory school						
Upper secondary school	0.95 (0.91 to 0.98)	0.93 (0.89 to 0.96)	0.91 (0.84 to 0.98)	0.98 (0.93 to 1.03)	0.91 (0.85 to 0.97)	0.89 (0.81 to 0.98)
Postsecondary education	0.92 (0.87 to 0.98)	0.86 (0.80 to 0.92)	0.81 (0.71 to 0.92)	0.98 (0.90 to 1.07)	0.93 (0.83 to 1.04)	0.86 (0.73 to 1.02)
Employment status, ref=employed						
Unemployed	1.15 (1.08 to 1.23)	1.46 (1.34 to 1.59)	1.42 (1.21 to 1.67)	1.05 (0.95 to 1.17)	1.26 (1.06 to 1.51)	1.12 (0.86 to 1.45)
Retired†	1.30 (1.24 to 1.37)	1.93 (1.82 to 2.04)	1.93 (1.72 to 2.16)	1.18 (1.10 to 1.27)	1.62 (1.45 to 1.81)	1.43 (1.23 to 1.68)
Profession, ref=upper white-collar						
Lower white-collar	1.00 (0.94 to 1.07)	1.00 (0.93 to 1.07)	1.04 (0.91 to 1.18)	1.01 (0.92 to 1.11)	1.03 (0.92 to 1.15)	1.07 (0.91 to 1.26)
Blue-collar	1.01 (0.96 to 1.06)	0.99 (0.94 to 1.04)	1.02 (0.93 to 1.12)	1.00 (0.94 to 1.07)	1.02 (0.94 to 1.11)	0.99 (0.88 to 1.12)
Others	1.05 (0.96 to 1.14)	1.14 (1.05 to 1.25)	0.98 (0.82 to 1.16)	1.00 (0.90 to 1.12)	1.18 (1.03 to 1.34)	1.13 (0.93 to 1.36)
Income per household member, ref=quartile 4						
Quartile 1	1.35 (1.27 to 1.44)	1.45 (1.35 to 1.56)	1.76 (1.54 to 2.02)	1.20 (1.11 to 1.30)	1.17 (1.05 to 1.30)	1.30 (1.11 to 1.53)
Quartile 2	1.25 (1.18 to 1.33)	1.34 (1.26 to 1.43)	1.44 (1.26 to 1.64)	1.13 (1.05 to 1.22)	1.15 (1.04 to 1.27)	1.23 (1.06 to 1.43)
Quartile 3	1.09 (1.03 to 1.16)	1.19 (1.11 to 1.27)	1.21 (1.05 to 1.38)	1.09 (1.01 to 1.17)	1.08 (0.98 to 1.19)	1.07 (0.92 to 1.24)
Diabetes medication, ref=yes						
No	1.12 (1.07 to 1.16)	1.09 (1.04 to 1.14)	1.09 (1.01 to 1.19)	1.09 (1.03 to 1.15)	1.03 (0.96 to 1.10)	1.07 (0.97 to 1.19)
Anticoagulants, ref=yes						
No	0.61 (0.59 to 0.64)	0.75 (0.73 to 0.78)	0.58 (0.54 to 0.63)	0.78 (0.73 to 0.83)	0.96 (0.89 to 1.03)	0.77 (0.68 to 0.86)
Antihypertensives, ref=yes						
No	0.87 (0.83 to 0.91)	0.93 (0.89 to 0.98)	0.85 (0.77 to 0.93)	0.93 (0.86 to 1.00)	1.02 (0.93 to 1.13)	0.98 (0.85 to 1.13)
Kidney disease, ref=no						
Yes	1.23 (1.13 to 1.34)	2.08 (1.96 to 2.21)	1.93 (1.72 to 2.16)	0.97 (0.84 to 1.11)	1.92 (1.75 to 2.11)	1.67 (1.46 to 1.92)
Cancer diagnosis‡, ref=no						
Yes	1.02 (0.94 to 1.11)	1.87 (1.76 to 2.00)	1.12 (0.96 to 1.30)	1.09 (0.98 to 1.21)	1.38 (1.23 to 1.55)	1.08 (0.90 to 1.30)
Diabetes duration						
Per year	1.01 (1.01 to 1.02)	1.01 (1.01 to 1.01)	1.02 (1.01 to 1.02)	1.01 (1.01 to 1.02)	1.01 (1.01 to 1.02)	1.02 (1.01 to 1.02)
HbA1c (mmol/mol), ref=below 42						
42–52	1.09 (1.03 to 1.15)	0.93 (0.88 to 0.99)	0.99 (0.88 to 1.12)	1.02 (0.94 to 1.10)	0.91 (0.82 to 1.01)	1.00 (0.86 to 1.17)
>52	1.29 (1.21 to 1.37)	1.10 (1.04 to 1.17)	1.27 (1.12 to 1.43)	1.21 (1.11 to 1.31)	1.10 (0.99 to 1.22)	1.25 (1.07 to 1.47)
eGFR, ref=60 or more						
<60 m	1.19 (1.13 to 1.24)	1.22 (1.17 to 1.28)	1.31 (1.21 to 1.42)	1.16 (1.10 to 1.23)	1.33 (1.25 to 1.42)	1.42 (1.29 to 1.56)
Cholesterol levels, ref=LDL <2.5, HDL <1.0 (men)/1.3 (women), triglycerides <2.0						
LDL ≥2.5	1.22 (1.17 to 1.26)	0.93 (0.90 to 0.97)	1.05 (0.98 to 1.13)	1.08 (1.03 to 1.14)	1.02 (0.96 to 1.08)	1.14 (1.04 to 1.24)
HDL ≥1.0 (men)/1.3 (women)	0.91 (0.88 to 0.95)	0.96 (0.92 to 1.00)	0.97 (0.90 to 1.05)	0.94 (0.89 to 0.99)	0.88 (0.83 to 0.94)	0.88 (0.80 to 0.97)
Triglycerides ≥2.0	1.09 (1.05 to 1.13)	1.00 (0.96 to 1.04)	1.07 (0.99 to 1.15)	1.03 (0.97 to 1.08)	0.91 (0.85 to 0.97)	0.97 (0.88 to 1.07)
BMI, ref=<25						
25–29	1.00 (0.95 to 1.05)	0.72 (0.69 to 0.75)	0.78 (0.71 to 0.85)	1.02 (0.96 to 1.09)	0.79 (0.73 to 0.85)	0.80 (0.71 to 0.89)
≥30	0.93 (0.89 to 0.98)	0.67 (0.64 to 0.70)	0.74 (0.68 to 0.82)	1.02 (0.95 to 1.10)	0.70 (0.65 to 0.76)	0.72 (0.64 to 0.82)
Blood pressure (mm Hg), ref=systolic <130, diastolic <80						
Systolic ≥130	1.14 (1.10 to 1.19)	0.84 (0.81 to 0.88)	0.93 (0.86 to 1.01)	0.96 (0.91 to 1.01)	0.81 (0.76 to 0.86)	0.80 (0.73 to 0.88)
Diastolic ≥80	0.97 (0.94 to 1.01)	0.93 (0.90 to 0.97)	0.92 (0.85 to 0.98)	0.97 (0.92 to 1.02)	0.96 (0.90 to 1.02)	0.97 (0.88 to 1.06)
Microalbuminuria, ref=no						
Yes	1.16 (1.11 to 1.21)	1.26 (1.21 to 1.31)	1.32 (1.22 to 1.42)	1.08 (1.02 to 1.14)	1.19 (1.12 to 1.27)	1.19 (1.08 to 1.31)
Macroalbuminuria, ref=no						
Yes	1.28 (1.21 to 1.36)	1.40 (1.32 to 1.48)	1.56 (1.42 to 1.72)	1.16 (1.07 to 1.25)	1.38 (1.27 to 1.50)	1.38 (1.23 to 1.56)
Physical activity§, ref=daily						
<1 time per week	1.25 (1.20 to 1.31)	1.79 (1.71 to 1.88)	1.85 (1.69 to 2.03)	1.17 (1.10 to 1.25)	1.72 (1.59 to 1.87)	1.74 (1.55 to 1.96)
1–2 times per week	1.10 (1.05 to 1.15)	1.26 (1.20 to 1.33)	1.30 (1.17 to 1.44)	1.05 (0.98 to 1.12)	1.18 (1.07 to 1.29)	1.19 (1.04 to 1.38)
3–5 times per week	0.99 (0.94 to 1.03)	1.04 (0.98 to 1.10)	0.98 (0.88 to 1.10)	1.00 (0.93 to 1.07)	1.05 (0.94 to 1.16)	1.04 (0.89 to 1.21)
Smoking¶, ref=no						
Yes	1.16 (1.11 to 1.22)	1.38 (1.31 to 1.44)	1.38 (1.26 to 1.51)	1.07 (1.00 to 1.14)	1.17 (1.07 to 1.28)	1.23 (1.08 to 1.41)

Adjusted HR (with 95% CI) for any CV events, all-cause mortality and CV mortality for patient characteristics among patients with type 2 diabetes mellitus by prevention type.

*Includes Belgium, Denmark, Germany, Greece, Spain, France, Ireland, Italy, Luxembourg, The Netherlands, Austria, Portugal, Finland, Sweden and Great Britain.

†If age 65 years or older and unemployed.

‡Within 5 years prior to baseline.

§30 min walk or equivalent.

¶At least one cigarette or pipe per day or quit smoking within 3 months.

BMI, body mass index;CV, cardiovascular;HDL, high-density lipoprotein;HR, hazard ratio;HbA1c, hemoglobin A1c;IQR, interquartile range;LDL, low-density lipoprotein;NA, not applicable;SD, standard deviation;TSEK, thousand Swedish Krona; eGFR, estimated glomerular filtration rate; ref, reference.

Primary prevention patients who had not filled prescriptions for antihypertensives had a lower risk of mortality unlike those who had not filled a prescription for diabetes medications that associated with increased risk. Furthermore, the risk of CV events and mortality was associated with lower income and unemployment. Among secondary prevention patients, increasing high-density lipoprotein cholesterol was associated with a lower risk of all three outcomes.

**Figure 2:**
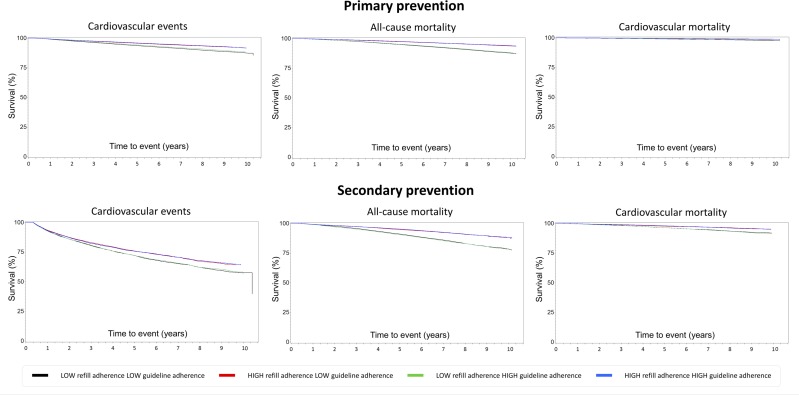
Adjusted Kaplan-Meier survival curves for cardiovascular (CV) events, all-cause mortality and CV mortality for patients with type 2 diabetes mellitus by prevention type.

Compared with low-adherent patients, the Kaplan-Meier survival curves showed higher survival of CV events and mortality among high-adherent patients (figure 2).

Primary prevention patients who were being treated at healthcare centers with low guideline adherence had lower survival of CV events than those who were being treated at high-adherent centers. Among high-adherent primary prevention patients, there was a tendency toward higher survival of CV events if they were being treated at high-adherent centers than at low-adherent centers. Among secondary prevention patients, guideline adherence had little or no impact on survival of CV events or mortality.

### Sensitivity analyses

When the refill adherence cut-off alternated between 60%, 80%, 70%, and 90%, or when the guideline adherence cut-off was set to 30% or 60% for primary prevention and 50% or 90% for secondary prevention, the risk of CV events and mortality showed a consistent pattern, that is, low-adherent patients faced a higher risk than high-adherent patients, independent of guideline adherence.

Adjusted for potential confounders, the risk of CV events and mortality was similar between complete cases and imputed data: low-adherent patients had a higher risk of CV events and mortality than high-adherent patients, independent of guideline adherence. Furthermore, low-adherent primary prevention patients who were being treated at low-adherent healthcare centers had a greater risk of an outcome than those treated at high-adherent healthcare centers. Additionally, in complete case analysis, low-adherent secondary prevention patients treated by low-adherent healthcare centers had lower risk of CV mortality compared with those treated by high-adherent healthcare centers.

## Discussion

In this nationwide study of more than 120 000 patients with T2DM, we analyzed the risk of CV events and mortality in relation to patient refill adherence to lipid-lowering medications and healthcare center adherence to lipid-lowering prescription guidelines. To our knowledge, this is the first study that considers both refill and guideline adherence when analyzing the risk of CV events and mortality among patients with T2DM.

We found that patients with low adherence to lipid-lowering medications had a higher risk of CV events and mortality in both prevention groups, independent of the center’s guideline adherence level. Furthermore, low-adherent primary prevention patients who were being treated at low-adherent centers faced a higher risk of CV events and CV mortality. Otherwise, guideline adherence had little or no effect on the risk of CV events or mortality in either prevention group. This suggests that patient adherence was the major factor with respect to lipid-lowering prevention of CV events and mortality among patients with T2DM.

Our results concur with previous studies that have shown that high refill adherence to lipid-lowering medications are associated with a lower risk of CV events and mortality than low refill adherence.^5 8 12 13^ Furthermore, we have previously shown that among multiple levels of refill adherence, the risk of CV events gradually increases with lower refill adherence.^6^ For those reasons, the healthcare center should be observant in order to detect early signs ofrefill non-adherence.

We found several patient characteristics associated with a higher risk of CV events and mortality in both prevention groups: male gender, increasing age, unemployment, lower income, higher HbA1c, lower kidney function, and so on. These characteristics have previously been shown to be associated with a higher risk of CVD and mortality in the general population^36^ and among patients with T2DM.^6^ Furthermore, we found patients without anticoagulants and antihypertensives, as well as those born outside of Sweden, to have lower risk of CV events and mortality. This differs from previous studies that have shown non-adherence to cardioprotective medications as associated with higher risk of CVD and mortality.^37 38^ However, a closer look reveals that patients without other cardioprotective medications and those born in Africa, the Americas, Oceania or Asia were younger, had shorter diabetes duration and less comorbidities, as well as shorter follow-up time for those born outside of Sweden. This could explain the lower risk of CV events and mortality in the present study. Our findings suggest that individual risk of CVD should be considered when choosing a diabetes treatment regimen.

Although adherence to lipid-lowering prescription guidelines had little or no impact on the risk of CV events and mortality, we have not considered nurses, dietitians, physical therapists, pharmacists, and so on, with whom patients with diabetes come in contact. Caregiver attitudes and beliefs concerning diabetes management have been shown to influence self-management behavior among patients with T2DM.^39 40^ Furthermore, patients with T2DM who had access to a diabetes team, group training programs and diabetes nurses had better control of their HbA1c.^40^

Low guideline adherence is not synonymous with low quality of care. In the present study, guideline adherence represented the prevalence of prescribed lipid-lowering medication among patients with T2DM with LDL cholesterol above the recommended target values entered in the NDR. We did not know why medications were not prescribed. Previous studies have shown that prescription of lipid-lowering medications for patients with T2DM is usually based on the individual risk of developing CVD rather than LDL cholesterol alone.^14 15 17–19^ Current treatment guidelines take a similar approach.^1 3 9 41^

### Strengths and limitations

The greatest strengths of this study are its national coverage and the linkage of individual data among national registers. By Swedish law, reporting to the National Patient Register and Cause of Death Register is mandatory; under-reporting is estimated at less than 1%. Thus, the results of this study tally well with clinical practice. Furthermore, the SPDR allowed an assessment of patient adherence using data about filled prescriptions instead of data about prescriptions that may or may not have been filled. However, we cannot be sure that patients actually took their medication, which constitutes the major limitation of our study.

As a result, we started following each patient when the first supply had ceased, that is, the first day of possible non-adherence. That eliminated the uncertainty concerning adherence that accompanies the first filled prescription, as it will always appear to be complete regardless of subsequent behavior.

Another limitation of our study is missing data, especially from the NDR. Not all patient characteristics are measured at every appointment, and most patients with T2DM have only an annual check-up. Nonetheless, the NDR includes data from both primary and specialized care clinics, permitting a population sample representative of patients in clinical practice. Furthermore, a sensitivity analysis between imputed data and complete cases showed similar results. Thus, the impact of missing data on our final conclusions appears to be minimal.

Since the National Patient Register does not cover diagnoses in primary care, only kidney disease diagnosed in inpatient or outpatient care was included. However, several other measurements of kidney function were accounted for (eGFR, microalbuminuria, macroalbuminuria, and so on). Thus, we believe that the study fully covered kidney disease as a risk factor.

Adverse drug reactions and contraindications would be legitimate reasons to abstain from prescribing lipid-lowering medications for patients with T2DM. However, no such information was considered. Thus, we may have included patients who were unsuitable for lipid-lowering medications. Furthermore, excluding patients who experienced CV events or died during their first filled supply or interval (n=15 425) may have introduced a selections bias that excluded the most fragile patients.

There was a statistically significant association between refill and guideline adherence. However, the association was too small to be clinically relevant. Thus, guideline adherence was assumed to have little or no impact on refill adherence and the variables were regarded as independent.

A comparison between the SPDR and the NDR regarding the healthcare center at baseline showed high conformity. The classification of type of care in the NDR was dichotomized as primary or specialized care, while the type of care in the SPDR was broken down into 19 categories. This may explain the lower agreement with respect to type of care.

## Conclusions

Patient refill adherence to lipid-lowering medications had a greater impact on the risk of CV events and mortality than healthcare center adherence to lipid-lowering guidelines or prevention type. These results demonstrate the value of individualized care among patients with T2DM, for example, through educational programs and risk factor counseling.

10.1136/bmjdrc-2018-000639.supp1 Supplementary data
