# Supplementary material for: Risk of cardiovascular event and mortality in relation to refill and guideline adherence to lipid-lowering medications among patients with type 2 diabetes mellitus in Sweden
Source: BMJ Open Diabetes Res Care. 2019 Apr 8;7(1):e000639. doi: 10.1136/bmjdrc-2018-000639 (PMC6501851; doi:10.1136/bmjdrc-2018-000639)
Supplement: Supplementary data [file bmjdrc-2018-000639supp001.pdf]

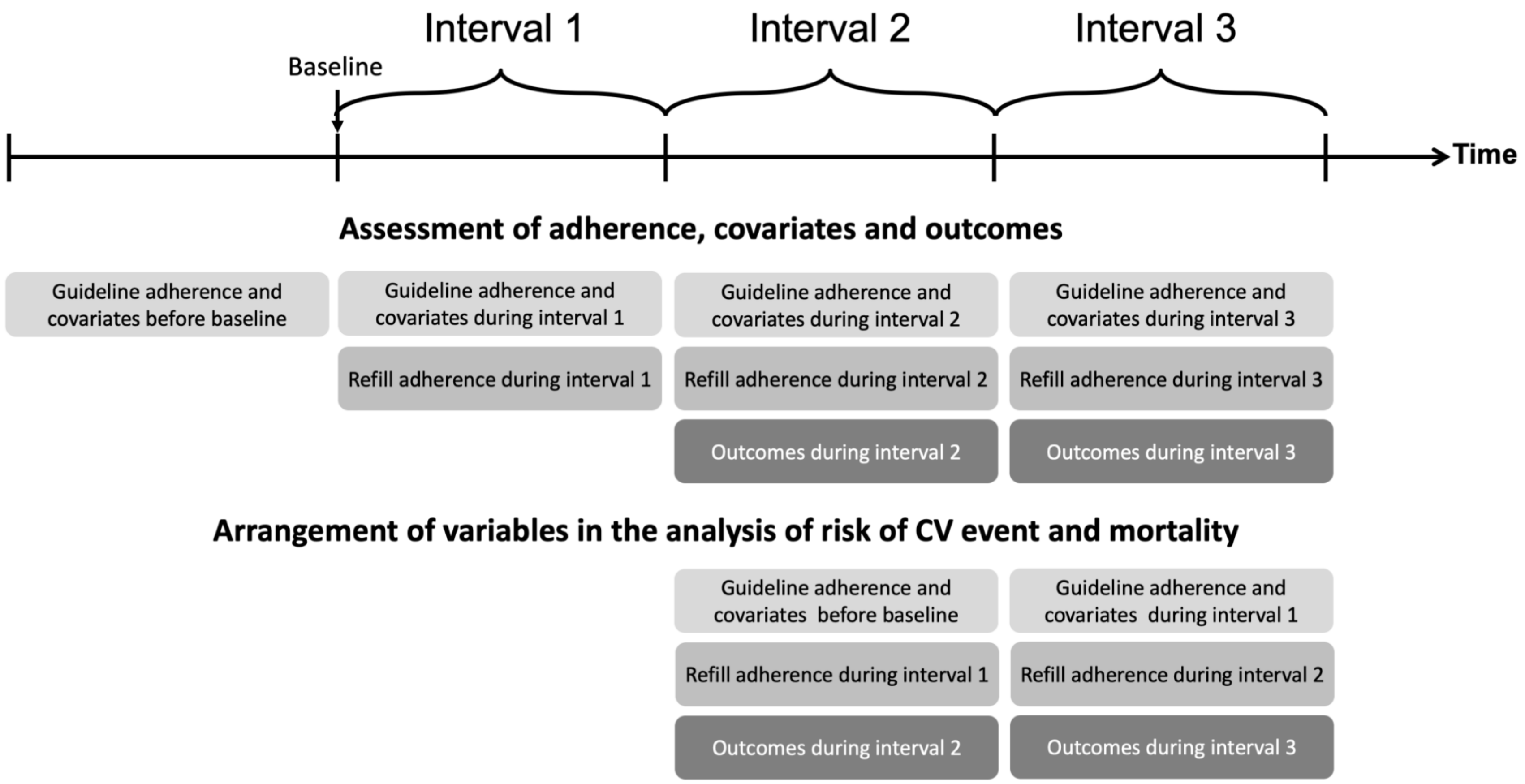

Figure S1. Assessment and arrangement of variables. Exposures, outcome and covariates were assessed for all intervals. In the analysis, covariates and guideline adherence for one interval were regarded as potential confounders for the subsequent interval of refill adherence measures. Similarly, refill adherence for one interval was regarded as the exposure for the subsequent interval of outcome measures.
